# Effects of Acupuncture Treatment in Reducing Sleep Disorder and Gut Microbiota Alterations in PCPA-Induced Insomnia Mice

**DOI:** 10.1155/2020/3626120

**Published:** 2020-10-29

**Authors:** Jiajing Hong, Jian Chen, Junming Kan, Mingjun Liu, Dongyu Yang

**Affiliations:** ^1^College of Acupuncture and Massotherapy, Changchun University of Chinese Medicine, Changchun 130117, China; ^2^Shanghai TCM-Integrated Hospital, Shanghai University of Traditional Chinese Medicine, Shanghai 200082, China; ^3^College of Chinese Medicine, Changchun University of Chinese Medicine, Changchun 130117, China; ^4^Affiliated Hospital, Changchun University of Chinese Medicine, Changchun 130117, China

## Abstract

Chronic insomnia without intervention will do harm to people's physical and psychological health as well as the quality of life. While ensuring efficacy, traditional Chinese medicine therapy, such as acupuncture, overcomes the side effects of drugs. However, the molecular mechanism of traditional medicine is unclear and it encounters many obstacles in repetitiveness and popularization. On the other side, the placebo effects also need to be eliminated during the intervention. In this study, a number of indicators such as duration of sleep latency, serum markers, pineal gland immunohistochemistry, and gut microbes were detected in the PCPA-induced insomnia mice to compare the effects between acupuncture and hypnotic drug treatments. Although the food intake and weight were not changed, the results show that serum maker and gut microbiota alterations were mediated by concurrent changes in sleep disorder induced by PCPA in mice. Compared with the PCPA-induced insomnia group, dopamine, 5-hydroxytryptamine, and norepinephrine were reduced in serum, and the melatonin was increased in the pineal gland of the acupuncture group as well as zopiclone drug group. Moreover, the analysis results from 16S tag sequencing of the gut microbiome bacterial rRNA hypervariable region show the same improvement effects between the two medical intervention groups. A co-occurrence network analysis showed that blank and acupuncture networks exhibited higher similarity than sham and zopiclone networks and the sham network possessed the highest complexity of microbial communities. Taken together, the gut microbiome will likely be a new target for improving sleep disorders, and taking into account the side effects of hypnotic drugs, nonpharmacological interventions such as acupuncture may be an effective means and have greater clinical benefits.

## 1. Introduction

Insomnia is the second most common mental disorder [1], associated with depression [2], cardiovascular disease [3], type 2 diabetes, and obesity [4], which affects 25–30% of adults worldwide [5]. It is characterized by difficulty starting or maintaining sleep, accompanied by symptoms like repercussions during wakefulness [6]. Currently, medications and nondrug therapies are used to treat primary insomnia. However, medications, such as melatonin and benzodiazepine, can cause adverse effects such as excessive neurological toxicity, addiction, and tolerance [7]. Cognitive behavioral therapy (CBT-I), an effective nondrug therapy, includes sleep hygiene, education about sleep, and relaxation techniques, which has been proven that it not only outranks hypnotic drugs with the short-term efficiency but also has long-term treatment efficiency [8]. However, due to high cost, lack of trained therapists, and intense labor, CBTs are not widely used, so that guidance for clinicians in choosing CBTs is limited so far [9]. Therefore, an increasing amount of individuals with insomnia are embracing complementary and alternative treatments such as acupuncture.

In China, acupuncture has been a popular nonpharmacologic modality and practiced for thousands of years. With stimulation of specific points (so-called “acupoints”) for “acu-,” and with penetrating the skin using thin, solid, metallic needles for “-puncture,” acupuncture provides effects mediated by regulation of neurotransmitters [10]. It is an alternative to prescription of medications [11] and often used in the oncology setting for pain management [12], musculoskeletal complaints, hot flashes, fatigue, stress, and anxiety [13]. Currently, acupuncture is commonly employed for handling mental wellness problems. Especially, many systematic reviews [7, 14, 15] and clinical randomized trials [16, 17] indicated that acupuncture could be effective against insomnia. Additionally, numerous studies have found that acupuncture can alter the composition of the gut microbiota [18–20].

Gut microbiome alteration is now claimed as relating to physiological sleep and has been identified as playing a significant role in the circadian misalignment according the accumulated evidence in the last decade [21–23]. In fact, changes in gut microbial communities have been mechanistically linked with nutritional absorption [24], metabolism and storage [25], as well as the establishment and maintenance of healthy immune [26] and metabolic functions [27]. The gut microbiota is affected by many factors, such as age, diet, and metabolic diseases, which are related to changes in the composition and function of the gut microbiota [28]. There is growing evidence illustrating that inflammation and stress caused by sleep disorders such as sleep apnea, sleep deprivation, sleep fragmentation, disordered circadian rhythm, and insomnia may be responsible for changes in the gut microbiota [29]. Moreover, a recent study has shown that the gut microbiota can be a subjective measurement for auxiliary diagnosis of insomnia [30]. However, it is unknown that changing the gut microbiome through acupuncture treatment might help treat insomnia or not.

To this end, we conducted a sham-controlled study to assess the benefits of acupuncture to improve sleep of PCPA-induced insomnia mice. The effects of sleep loss and improvement on the gut microbiota were evaluated and combined with a number of indicators to verify whether acupuncture therapy has similar efficacy with hypnotics.

## 2. Materials and Methods

### 2.1. Animals

C57BL/6 male mice (6 weeks old), weighing 20.00 ± 2.0 g, were purchased from the Model Animal Research Center (Nanjing, Jiangsu, China). The animals were acclimatized to an ambient temperature of 25 ± 2 °C, relative humidity (50 ± 5%), and a 12/12-h light-dark cycle for 2 weeks and received a standard chow diet throughout the experiment. The experimental procedure and protocol were approved by and performed in the Laboratory Animal Center of Changchun University of Chinese Medicine according to the National Guidelines for Experimental Animal Welfare (Ministry of Science and Technology of the People's Republic of China, 2006).

### 2.2. Mice Grouping

**Figure 1:**
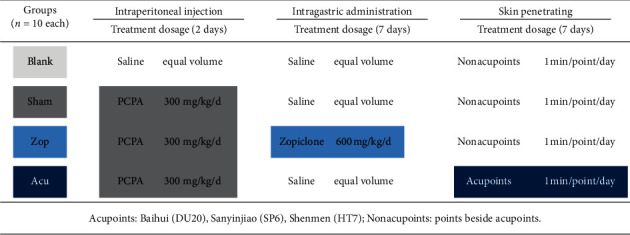
Grouping information of mice and corresponding intervention methods. Four groups of mice model were named blank, sham, Zop, and Acu, respectively. At 2 days after PCPA injection, zopiclone and acupuncture treatments were administered for 7 days. Normal saline was sham controlled for drugs such as PCPA and zopiclone. Penetrating on nonacupoints was sham controlled for acupuncture on acupoints (DU20, SP6, and HT7).

Mice were randomly divided into four groups (10 mice for each group): blank, sham, Zop, and Acu with different treatments as shown in Figure 1.

### 2.3. p-Chlorophenylalanine- (PCPA-) Pretreated Mouse Model

For the PCPA-induced insomnia model, mice received intraperitoneal injection of PCPA (300 mg/kg) between 08 : 00 and 09 : 00 once a day for 2 days [31, 32]. After the second intraperitoneal injection of PCPA for 28 h, the mouse model showed the disappeared circadian rhythm, continued daytime activities, enhanced excitability, increased aggression and urine and stool production, loss of appetite, and development of dull and dishevelled hair and grey stools. The blank group received intraperitoneal injection of the same volume of weakly alkaline saline.

### 2.4. Zopiclone Treatments

In the hypnotic treatment group, which is named Zop group, zopiclone was formulated into a solution and was administered intragastrically with the dosage of 0.6 mg/kg. The nondrug groups were given the same volume of normal saline.

### 2.5. Acupuncture Treatment

In the acupuncture treatment group, which is named Acu group, according to the acupoint spot shown in the mouse acupoint map, mice were treated with acupuncture on the acupoints of Baihui (DU20), Sanyinjiao (SP6), and Shenmen (HT7). The needle was evenly inserted 1 mm depth and twisted for 1 min at each acupoint. The other groups were given the same stimulation but not on the acupoints.

### 2.6. Subhypnotic Dosage of Sodium Pentobarbital

Sodium pentobarbital (30 mg/kg) was administered 50 min after different treatments, and the onset of sleep was observed in each mouse group. Mice were considered to be asleep when they lost righting reflex lasting more than 1 minute. Then, we calculated the sleep latency (the time between injection of pentobarbital sodium and disappearance of righting reflex) and the sleep duration (the time between disappearance of righting reflex and the time when the mouse woke up). Finally, all the mice were fasted overnight before blood testing and tissues collection. On day 8, all the mice were sacrificed 2 h after administration by cervical dislocation under deep chloral hydrate anesthesia, to collect biological materials for the following test.

### 2.7. Determination of DA, 5-HT, and NE Levels

The levels of DA (dopamine), 5-HT (5-hydroxytryptamine), and NE (norepinephrine) concentrations from mice serum were quantified by specific ELISA sandwich assays, performed using an ELISA kit (Abcam, Cambridge, UK) according to the manufacturer's protocol. Absorbance was read at 450 nm using a microtiter plate reader (PerkinElmer, Inc., Waltham, MA, USA).

### 2.8. Immunohistochemistry of Melatonin

Brain tissues of mice were ﬁxed, embedded, and sectioned to detect melatonin changes in the pineal gland. After dewaxing and rehydration, the sections were pretreated with peroxidase blocking buffer for 20 minutes and 5% normal goat serum in phosphate-buffered saline for 1 hour at room temperature, and then the sections were incubated with an anti-Melatonin antibody (1 : 500, overnight at 4°C; Abnova) in the blocking buffer. The secondary antibody reagents were from a 3,3'-diaminobenzidine tetrahydrochloride kit.

### 2.9. 16S rRNA Gene Tag Analysis

Fecal DNA was isolated using a Stool Fast Mini kit (Qiagen). The 16S rRNA tag libraries were generated using the set of indexed primers (V3–V4 hypervariable region) and sequenced on the Illumina MiSeq platform for generating 2 × 250 bp paired-end reads, processed, and annotated vs. the RDP database (v.9) using Mothur software. Metastats software was used to determine differentially abundant taxa; PCoA was used to compare group points. The protein sequences of genes in the merged gene catalogue were aligned to the Clusters of Orthologous Groups (COG) and the Kyoto Encyclopedia of Genes and Genomes (KEGG) database through PICRUSt.

### 2.10. Network Analysis

Network analysis was conducted separately for each group by following the Molecular Ecological Network Analyses (MENA) Pipeline (http://ieg4.rccc.ou.edu/mena/) [33]. Network was created based on Pearson correlation. In addition, the following options were selected: OTUs detected in more than 5 samples were used; 0.01 was filled in the blanks; calculation was executed by decreasing the cutoff from the top with a Poisson regression only; and a similarity threshold (cutoff = 0.9) was selected by the random matrix theory (RMT)-based approach to construct the network. Subsequently, modules were calculated using fast greedy modularity optimization. Finally, topological roles of each node were examined calculating Zi (within-module connectivity) and Pi (among-module connectivity). Calculated networks were visualized using Gephi [34].

### 2.11. Statistical Analysis

All values are expressed as mean ± standard deviation (SD). Analyses of variance procedures followed by post hoc tests, and Student *t*-tests were used to compare the results between blank, sham, Zop, and Acu groups. In all cases, a two-tailed *P*-value of <0.05 was considered to achieve statistical significance.

## 3. Results

### 3.1. Acupuncture Significantly Improved Physiological Phenotypes and Sleep Onset in Insomnia Mice

**Figure 2:**
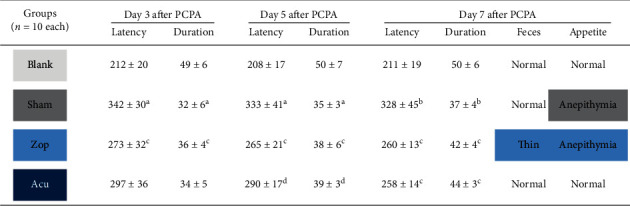
Effect of treatments on the sleep onset and physiological phenotype. The latency (seconds) and duration time (mins) of sleep onset in mice treated with a subhypnotic dose of sodium pentobarbital were expressed as mean ± standard error of the mean. *aP* < 0.001 and b*P* < 0.01 vs. the blank group; c*P* < 0.01 and d*P* < 0.05 vs. the sham group. Feces and appetite status were recorded daily, and status summaries were generated at the last day of treatment.

To investigate the effects of treatment on the development of insomnia in mice, the mice with insomnia were divided into groups received intervention including zopiclone (Zop group) and acupuncture (Acu group) (*n* = 10 each group, see Figure 1). In control group (blank group), the mice had bright hair color, normal breathing and circadian rhythm, normal feces, and stable appetite. On the contrary, mice from the PCPA-induced insomnia group (sham group) had obvious mania, disordered circadian rhythm, dim hair color, tachypnea, and loss of appetite. Unsatisfactorily, the circadian rhythm of Zop mice was gradually restored, but their feces were sparse and appetite was depressed, which may be side effects of drugs. In the acupuncture group, after acupuncture treatment lasting 7 days, circadian rhythm, hair color, breathing, feces shape, and appetite had recovered (Figure 2). Furthermore, after subhypnotic dosage of pentobarbital, the results of latency and duration time of sleep revealed that the acupuncture group exerted the similar hypnotic sedative effects as zopiclone drugs in improving sleep of PCPA-induced insomnia mice.

### 3.2. Effects of Acupuncture on Hormone Recovery in Insomnia Mice

**Figure 3:**
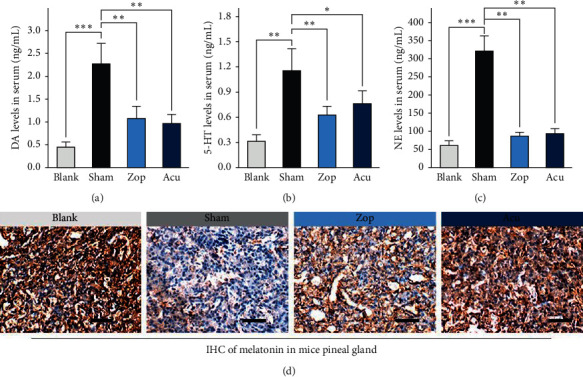
Effects on the hormone level changes in the serum and pineal body. (a-c) Effects on the concentration of (a) DA, (b) 5-HT, and (c) NE in the serum of four mice groups (*n* = 10 each). Data are expressed as a mean ± standard error of the mean. ^*∗*^*P* < 0.05, ^*∗∗*^*P* < 0.001, and ^*∗∗∗*^*P* < 0.001. (d) Representative immunostaining images of melatonin in mice pineal body tissue. The decreased level of melatonin in the sham group was recovered in Zop and Acu groups. Scale bars: 100 *μ*m.

The levels of dopamine (DA), 5-hydroxytryptamine (5-HT), and norepinephrine (NE) in serum were detected to investigate the effects of acupuncture on insomnia improvement. Notably, the levels in the Zop group and the Acu group were decreased significantly compared with those in the sham group in which hormones increased abnormally (Figures 3(a)–3(c)). Furthermore, the decreasing levels of melatonin in the pineal body were significantly recovered in the Zop and the Acu group (Figure 3(d)).

### 3.3. Sequencing and Quality Control of Gut Microbiota

**Figure 4:**
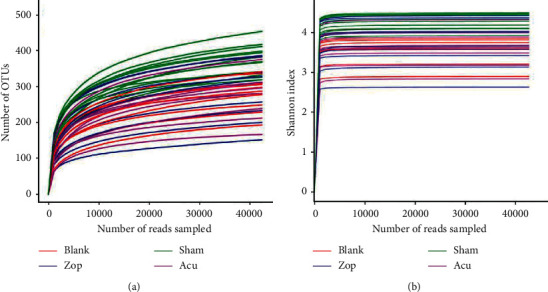
Curves tended to approach the saturation plateau. (a) Rarefaction curves for OTUs and (b) Shannon index of the OTU number at 97% similarity. Red, green, blue, and purple colors represent blank, sham, Zop, and Acu groups, respectively. Tags were normalized to the minimum numbers of tags in all samples.

The total clean reads of the 40 samples (*n* = 10 for 4 groups including blank, sham, Zop, and Acu) were 3,486,200 after filtering and 45,001–129,559 sequences per sample (mean = 87155). The sequencing analysis of the 40 samples identified 885 OTUs. The coverage indexes for all samples were greater than 0.99, which suggested that the sequencing capability was large enough to capture the majority of the diversity for each sample. The rarefaction curves (Figure 4(a)) tended to approach the saturation plateau, indicating that the number of samples used in this study was reasonable. The same tendency was found in the Shannon–Wiener curves (Figure 4(b)), indicating that the database of the V3–V4 hypervariable region from 16S rRNA gene sequences reflected the vast majority of microbial information.

### 3.4. Diversity Indices of Microbiota

**Figure 5:**
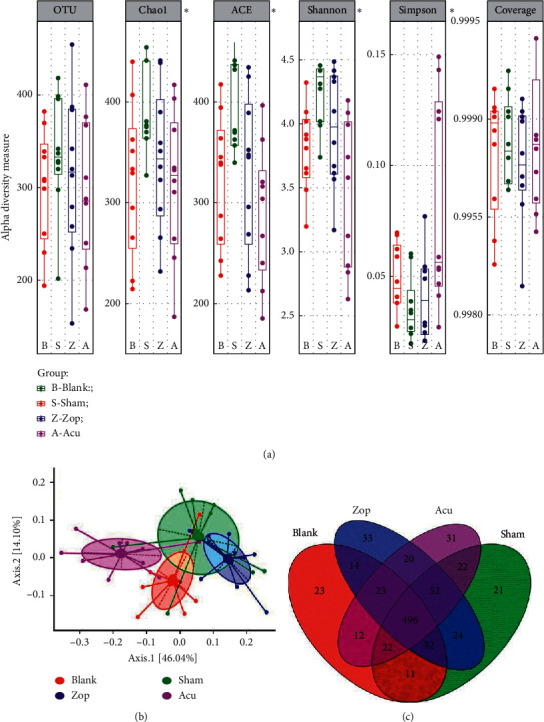
Diversity indices of gut microbiota. (a) Estimated OTU richness and diversity indices (Chao1, ACE, Shannon, and Simpson) of the mice gut microbiome from four groups. An asterisk indicates index with signiﬁcant differences (*P* < 0.05, one-way ANOVA). (b) Principal coordinate analysis (PCoA) of gut microbiota from four groups. Axis 1 explains the greatest proportion of compositional variation followed by Axis 2. (c) Venn plot showing the shared and unique OTUs found in each plotted group. Only taxa that are shared by all of the animals (core) within each group are plotted. Red, green, blue, and purple colors represent blank, sham, Zop, and Acu group, respectively.

The indices (OTUs, Chao1, ACE, Shannon) showed that the sham group exhibited higher bacterial diversity than blank group, and both treatments reduced the OTU richness (Figure 5(a)). The data of four groups were further analyzed by PCoA (principal coordinate analysis) to identify the overall difference in the gut microbial community among different treatments.  Figure 5(b) shows a distinct separation between these four groups, and the first axis could explain variation for different groups. Besides, the distances of blank group, Zop group, and Acu group were getting close on PC2, which reminds that the three groups had a relative similar community structure compared with the sham group (Figure 5(b)). However, there are unique OTU in different groups, which suggests that the bacterialcommunities are different and change rapidly during the seven days of treatment (Figure 5(c)).

### 3.5. Key Phylotypes of Gut Microbiota Modulated by Acupuncture

**Figure 6:**
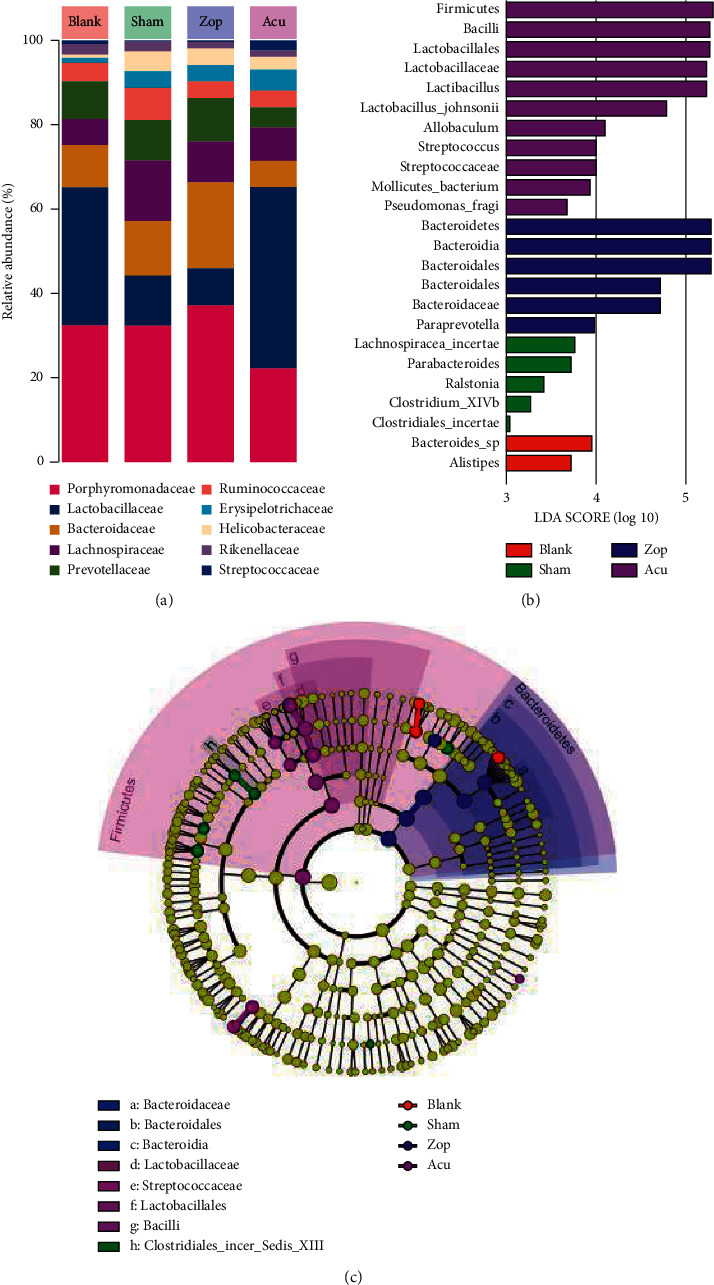
Key phylotypes of gut microbiota modulation. (a) Gut microbiota of four group taxa summaries (presented at the family level) of bacterial relative abundance. (b) Indicator microbial groups within the four groups with LDA values higher than 3. (c) Cladogram indicating the phylogenetic distribution of microbial lineages associated with the four groups; lineages with LDA values of 3 or higher determined by LEfSe are displayed. Red, green, blue, and purple colors represent blank, sham, Zop, and Acu groups, respectively.

**Figure 7:**
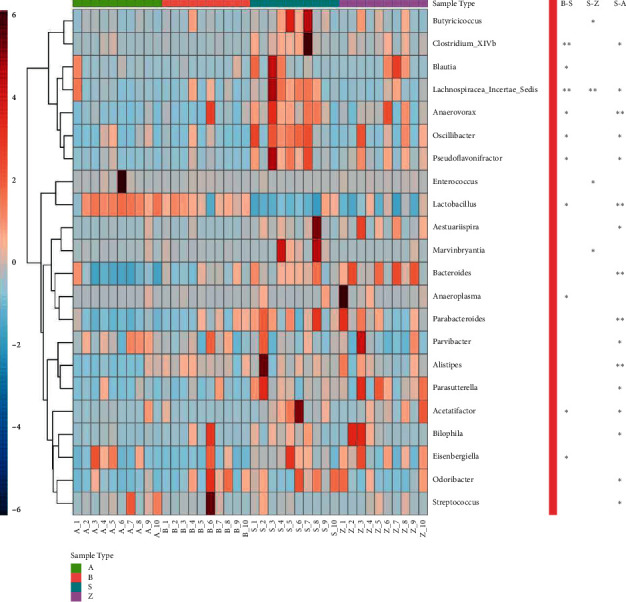
The heatmap of the relative abundance of microbes influenced by acupuncture treatment at the genus level. The firebrick color means high abundance, while the navy color means low values. ^*∗∗*^*P* < 0.01 and ^*∗*^0.01 ≤ *P* < 0.05. B, blank; S, sham; Z, Zop; A, Acu.

Therefore, we examined each group separately. At the family level, a substantial decrease in the family *Bacteroidaceae* and an increase in the family *Lactobacillaceae* were observed (Figure 6(a)) As an algorithm to robustly identify features that are statistically different among groups, linear discriminant analysis (LDA) effect size (LEfSe) was employed to identify speciﬁc phylotypes responding to PCPA-induced insomnia and zopiclone or acupuncture treatment. Overall, *Lachnospiracea incertae sedis*, *Clostridiales incertae sedis*, and *Parabacteroides* were significantly increased in the sham group, indicating that the insomnia disorder could significantly alter the population of the gut microbiota. Moreover, several lineages had an LDA value of 5 or higher, namely, *Firmicutes*, *Bacilli*, *Lactobacillus*, and *Bacteroidales*. It showed that the *Firmicutes*/*Bacteroidetes* ratio of gut microbiota in the Acu group was increased compared with the other three groups. Apparently, *Bacteroides, Lachnospiracea incertae, Bacteroidetes,* and *Firmicutes* were the most abundant differential microbiota in the blank, sham, Zop, and Acu treatment groups, respectively (Figures 6(b) and 6(c)). Subsequently, 2D clustering was performed to further identify the alterative distribution of the fecal microbial community in the mice at the genus level (Figure 7). Generally, the gut microbiome alterations in the blank and acupuncture groups were similar, while the gut microbiome changes in the sham and zopiclone groups were similar. Specifically, as compared with the blank group, the relative abundance of *Clostridium XlVb*, *Lachnospiracea incertae sedis*, *Anaerovorax*, *Oscillibacter, Pseudoflavonifractor,* and *Acetatifactor* was increased in the sham group, while the relative abundance of these bacteria in the acupuncture treatment group was less than the sham group. Besides, *Lactobacillus* possessed the opposite trend in alteration.

### 3.6. Functional Analysis

**Figure 8:**
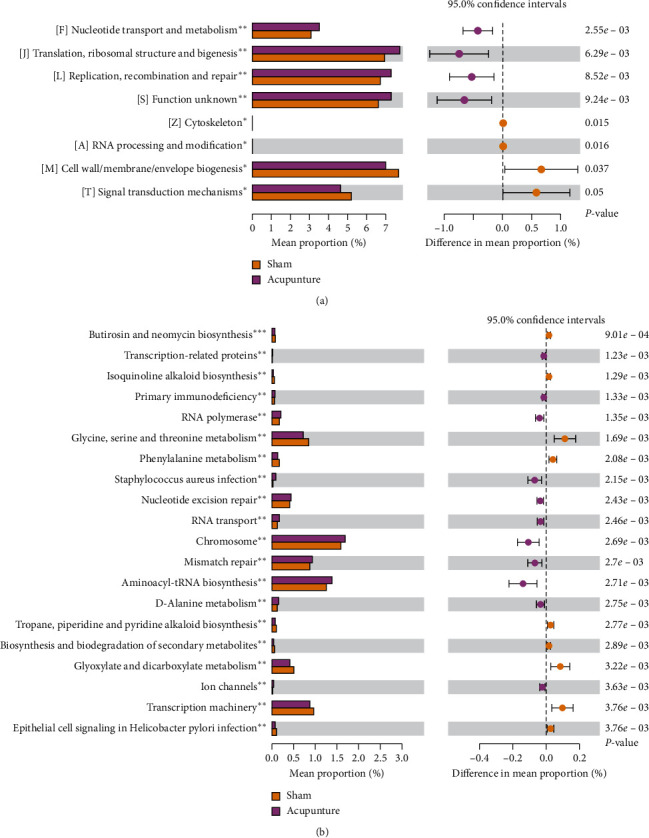
Distribution of COG (a) and the top 20 KEGG (b) functional category markers between the sham and acupuncture groups.

PICRUSt was used to annotate the gene catalogue by COG and KEGG modules. We found 8 and 124 differently abundant terms at COG and KEGG levels, respectively, which suggested a diverse change in the functions of the microbiota in the acupuncture group compared with shams (Figure 8). For COG terms, the upregulated COGs in the acupuncture group were nucleotide transport and metabolism, replication, recombination, and repair, while the downregulated COGs were cytoskeleton, RNA processing and modification, and cell wall/membrane/envelope biogenesis. For KEGG terms, the upregulated pathways in the acupuncture group were transcription-related proteins, primary immunodeficiency, and RNA polymerase, while the downregulated pathways were butirosin and neomycin biosynthesis, isoquinoline alkaloid biosynthesis, glycine, serine, and threonine metabolism.

### 3.7. Complexity of the Microbial Network among the Four Groups

**Figure 9:**
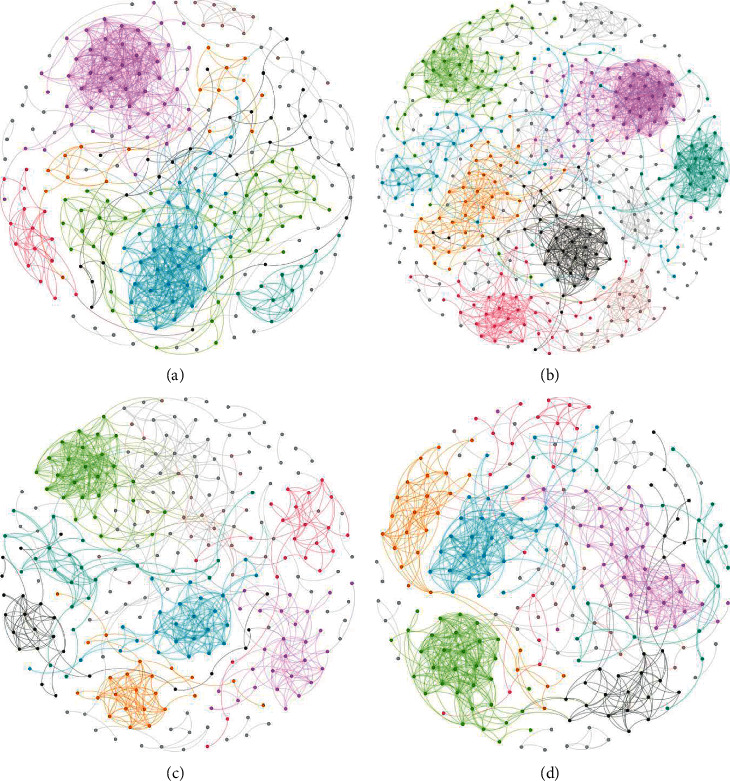
The co-occurrence pattern structure of microbial networks in the blank (a), sham (b), Zop (c), and Acu (d) based on the fast greedy modularity optimization method. Nodes represent microbial OTUs, and the color denotes different modules.

Potential interactions between microbial taxa in the four groups were explored using a RMT-based network analysis.  Table 1 shows the topological properties of the networks of microbial communities in the blank, sham, Zop, and Acu groups. The network connectivity of the four networks had a scale-free properties with an R2 value greater than 0.6. In the sham network, the values of properties including the number of nodes, average path distance (GD), harmonic geodesic distance (HD), maximal betweenness, centralization of betweenness (CB), centralization of stress centrality (CS), connectedness (Con), and modularity were highest, but the average degree (avgK), geodesic efficiency (E), centralization of degree (CD), density (D), and transitivity (Trans) were lower than that in the blank and Acu network. These findings indicated that microbial communities in the sham group formed more complex networks than those in other 3 groups (Figure 9). In addition, OTU83 (*Porphyromonadaceae*) and OTU153 (*Saccharibacteria genera incertae sedis*), OTU160 (*Acetatifactor*), OTU77 (*Lachnospiraceae*), and OTU43 (*Lachnospiraceae*) showed the highest degree for blank, sham, Zop, and Acu networks, respectively. OTU184 (*Lachnospiraceae*), OTU66 (*Clostridium IV*), OTU457 (*Ruminococcaceae*), and OTU268 (*Firmicutes*) showed the highest betweenness for blank, sham, Zop, and Acu networks, respectively.

## 4. Discussion

The acupuncture treatment on classic acupoints has a long history of its effects in relieving and improving sleep quality [35]. Given the challenge of this complex therapeutic system for treating insomnia using acupuncture, a more complete and systematic research is necessary to further guide future clinical and research directions [9]. We conducted a series of research on insomnia mice, using the measures related to sleep disorder including observation of behavior, sleep onset, hormones in serum and brain tissue, and especially gut microbiome alterations. To eliminate the placebo effects, the treatments of normal saline or puncture on nonacupoints were used to discriminate improvements from sham controls. The primary objective of this study is to demonstrate the acupuncture on the acupoints of Baihui (DU20), Sanyinjiao (SP6), and Shenmen (HT7) in the insomnia mice model and to provide theoretical support and data reference for subsequent research and clinical treatment.

Baihui (DU20), Sanyinjiao (SP6), and Shenmen (HT7) acupoints are historically used for treating insomnia [36]. Our findings showed that stimulating the combination of these acupoints can show effects on improving sleep in an experimental mice model of insomnia. These findings are consistent with previous research [31]. However, the mechanism of specific acupoint stimulation and acupoint combination need to be further studied. A recent study has found that electroacupuncture at the Baihui (DU20) acupoint shows neuroprotective effects via inhibiting the apoptosis of nerve cells, decreasing the abnormally level of *β*-amyloid-42, and increasing BDNF expression in APP/PS1 transgenic mice [37]; acupunctureat the Sanyinjiao (SP6) has a role in painless childbirth, in the treatment of urinary and reproductive disorders, and as anesthesia during pelvic surgery [38]; the HT7 (Shenmen) acupuncture point can ameliorate mental disorders, such as depression, anxiety, and drug addiction [39]. Importantly, another survey has found that acupuncture at the Baihui (DU20) and Sanyinjiao (SP6) had a role in ameliorating ischemic stroke through neuroprotective effects with anti-inflammation and TRPV-1-mediated antioxidant stress function [40]. Overall, the treatment of insomnia using acupuncture stimuli on Baihui (DU20), Sanyinjiao (SP6), and Shenmen (HT7) may be related to the combined actions of the immune system and the central nervous system. Therefore, it is particularly important to further study its mechanism.

Next, we focused on the changes of gut microbiota on insomnia mice. Recently, dysregulation in gut microbiota has involved in the pathogenesis of mental disorders such as insomnia and depression [41]. The bidirectional link between gut microbiota and mental disorders is becoming increasingly apparent. First, increasing studies revealed that mental disorders have an influence in the metabolism of indigenous gut bacteria and trigger microbial dysbiosis [42, 43]. Second, gut microbial dysbiosis may induce the abnormal immune reactions, which further affect the central nervous system, inducing or aggravating depression and insomnia [44, 45]. In the present study, we found that *Clostridium XlVb*, *Lachnospiracea incertae sedis*, *Anaerovorax*, *Oscillibacter, Pseudoflavonifractor,* and *Acetatifactor* were the microorganisms with lower abundance in acupuncture treatment and blank groups, compared with the sham group, while *Lactobacillus* possessed the opposite trend in alteration. Moreover, the LEfSe analysis showed that a variety of intestinal genera and species were enriched in blank, sham, zopiclone, and acupuncture groups, whereas the log LDA scores of *Firmicutes* in the acupuncture group, *Bacteroidetes* in the zopiclone group, *Lachnospiracea incertae* in the sham group, and *Bacteroides* sp. in the blank group were the highest. Interestingly, our co-occurrence network analysis also showed that blank and Acu networks exhibited higher similarity than sham and Zop networks; and the sham network possessed the highest complexity of microbial communities, as shown by the greater number of nodes and links, higher GD value, connectedness, and modularity. Taken together, the results indicated that the insomnia mice had more complicated microbe-microbe interactions. *Lachnospiracea incertae sedis*, a butyrate-producing bacterium, can exert an anti-inflammatory function by triggering regulatory T cells, which further regulate the immune system [46]. *Lactobacillus* can decrease levels of inflammatory cytokines and combat the TLR-related inflammation in chronic sleep fragmentation mice [47]. *Oscillibacter* abundance was considerably positively related to IL-1*β* and IL-6 expressions and ulcerative colitis pathological scores in mice [48]. Together with the observation on the immune regulation role of acupuncture [49, 50], we concluded that acupuncture treatment may have therapeutic effects on insomnia through regulating the gut flora to modulate the host immune response.

More and more evidence had proved that gut microbiome alterations are closely related to the sleep status of rats, mice, and people. However, there remains a large gap in the mechanisms of them. For example, the change of *Firmicutes/Bacteroidetes* ratio of gut microbiota, is it a result or a reason for sleep disorder? Is the alteration of gut microbiota a symptom or a therapeutic target? Somehow, the causal relationship is still stuck in a black box. In this study, besides the observation of change in diversities and communities, we also found that upregulated *Bacteroidales* in the hypnotic treatment group; and it is noteworthy that the side effect due to short-term relief from insomnia using hypnotic drugs such as abnormal appetite may be caused by gut microbiome disorder. Nevertheless, it will also raise novel questions regarding the mechanisms by which gut microbiota interact with the host, and whether these gut microbiome alterations are causal risk factors of insomnia or just reflect the acupuncture therapy. Many researchers believed that the microbiota-brain-gut axis can explain their relationship [30, 41]. The gut microbiome alterations may influence the brain function through immune-neuroendocrine or vagal stimulation mechanism, respectively [51, 52]. Current studies have revealed that acupuncture mediated gut microbiota through brain-gut peptides [53]. Collectively, these findings suggest a potential role of the brain-gut axis in increasing appetite and ameliorating insomnia during acupuncture therapy.

## 5. Conclusions

This study confirmed that compared with the placebo group and the hypnotic group, acupuncture stimulation on Baihui, Sanyinjiao, and Shenmen acupoints helps improve the sleep disorder phenotype in animal models and has a significant effect on gut microbiota recovery.

## Figures and Tables

**Table 1:** Topological properties of the networks of microbial communities in the four groups.

Network indexes	Blank	Sham	Zop	Acu
Total nodes	258	318	280	244
Total links	1017	1094	844	930
R square of power-law	0.709	0.616	0.775	0.663
Average degree (avgK)	7.884	6.881	6.029	7.623
Average clustering coefficient (avgCC)	0.462	0.493	0.472	0.551
Average path distance (GD)	5.913	10.13	6.99	6.265
Geodesic efficiency (E)	0.251	0.172	0.213	0.233
Harmonic geodesic distance (HD)	3.988	5.805	4.687	4.295
Maximal degree	33	23	22	24
Nodes with max degree	OTU83; OTU153	OTU160	OTU77	OTU43
Centralization of degree (CD)	0.098	0.051	0.058	0.068
Maximal betweenness	3981	13430.34	5764.954	5149.367
Nodes with max betweenness	OTU184	OTU66	OTU457	OTU268
Centralization of betweenness (CB)	0.109	0.246	0.138	0.162
Centralization of stress centrality (CS)	7.113	30.109	1.132	3.714
Maximal eigenvector centrality	0.218	0.249	0.258	0.25
Centralization of eigenvector centrality (CE)	0.197	0.233	0.241	0.23
Density (D)	0.031	0.022	0.022	0.031
Transitivity (trans)	0.711	0.635	0.678	0.69
Connectedness (con)	0.624	0.799	0.534	0.633
Number of modules	22	26	27	17
Modularity	0.697	0.82	0.82	0.796

## Data Availability

The data are available upon request to the corresponding author.

## Abbreviations

PCPA: p-Chlorophenylalanine
DA: Dopamine
5-HT: 5-hydroxytryptamine
NE: Norepinephrine
Sham: Puncture treatments on non-acupoint
Zop: Zopiclone treatment
Acu: Acupuncture treatment
DU20: Baihui acupoint
SP6: Sanyinjiao acupoint
HT7: Shenmen acupoint
IHC: Immunohistochemistry
OTUs: Operational taxonomic units
ACE: Abundance-based coverage estimator
PCoA: Principal coordinate analysis
LDA: Linear discriminate analysis
LEfSe: LDA effect size
COG: Clusters of Orthologous Groups
KEGG: Kyoto Encyclopedia of Genes and Genomes
Zop: Zopiclone
Acu: Acupuncture.
